# Case-control analysis in resting and evoked inflammatory profiles

**DOI:** 10.1186/1744-8069-10-S1-P3

**Published:** 2014-12-15

**Authors:** Christopher King, Margarete Ribeiro-Dasilva, Roger Fillingim, Shannon Wallet

**Affiliations:** 1 Pain Research and Intervention Center of Excellence (PRICE), University of Florida, Gainesville, FL 32610, USA; 2 Periodontology and Oral Biology, University of Florida, Gainesville, FL 32610, USA

## Background

While case-control differences in inflammation have been reported in several chronic pain conditions, the question of whether Temporomandibular Disorder (TMD) is also characterized by a heightened pro-inflammatory profile has not been investigated thoroughly. Inflammatory profiles can be evaluated by a number of methods including measuring basal (resting) levels and reactivity following a painful stressor. Therefore, the objective of the study was to evaluate case-control differences in a) resting levels of inflammation and b) impact of experimentally induced pain on inflammatory responsivity. The current study tested the hypothesis that individuals with TMD will show a greater pro-inflammatory profile at rest and following pain induction compared to controls. Exploratory analysis of the associations between inflammation and clinical outcomes were also conducted.

## Materials and methods

Individuals with (n=9) and without (n=20) TMD were recruited from the University of Florida. Blood was collected in EDTA tubes before and up to 90 minutes following an experimental testing procedure including heat (forearm, cheek) and cold immersion (foot). Blood was placed on ice, processed, and stored at -80°C. Simultaneously measurement of pro- (TNFα, IL-6, IL-8) and anti- (IL-4, IL-5, IL-10) inflammatory levels was performed with multiplex kits (Millipore). A visual analog scale (VAS) was used to measure cold and heat pain. To assess reactivity, area under the curve with respect to increase (AUCi), which controls for baseline values, was assessed for each cytokine across time. Group differences in variables derived from resting and reactivity measures were evaluated as dependent variables in separate analyses of covariance controlling for age, menstrual cycle, and time of collection. Associations between resting inflammatory levels, clinical measures of pain and disability were assessed with partial correlations.

## Results

Markers of resting inflammation were higher in the TMD group (all *p’s* < .05) and positively associated with clinical measures (all *p’s* < .05). While no case-control differences were observed in pain sensitivity, an increase in inflammation was observed in both groups following pain induction. Compared to the control group, pro-inflammatory markers were significantly higher (all *p’s* < .001) while a trend was observed for lower levels of anti-inflammatory markers (all *p’s* < .10) in individuals with TMD.

## Conclusions

The current study suggests that individuals with TMD may exhibit a pro-inflammatory imbalance (i.e., enhanced release of pro-inflammatory markers; blunted release of anti-inflammatory markers), which may contribute to the clinical phenotype. Additional research is needed to determine the significance of these findings.

## Disclosures

None.

